# Association of variants of miRNA processing genes with cervical precancerous lesion risk in a southern Chinese population

**DOI:** 10.1042/BSR20171565

**Published:** 2018-05-28

**Authors:** Shi-Qi Huang, Zi-Xing Zhou, Shao-Ling Zheng, Dan-Dan Liu, Xiao-Hong Ye, Cheng-Li Zeng, Ya-Jing Han, Zi-Hao Wen, Xiao-Qian Zou, Jing Wu, Yu-Mei Liu, Chui-Can Huang, Yao Wang, Guang Yang, Chun-Xia Jing

**Affiliations:** 1 Department of Epidemiology, School of Medicine, Jinan University, No.601 Huangpu Road West, Guangzhou 510632, Guangdong, China; 2 Department of Pathogen Biology, School of Medicine, Jinan University, Guangzhou 510632, Guangdong, China; 3 Guangzhou Key Laboratory of Environmental Exposure and Health, Guangdong Key Laboratory of Environmental Pollution and Health, Jinan University, Guangzhou 510632, Guangdong, China

**Keywords:** Cervical precancerous lesions, interactions, miRNA processing genes, single nucleotide polymorphisms

## Abstract

The miRNA processing genes play essential roles in the biosynthesis of mammalian miRNAs, and their genetic variants are involved in the development of various cancers. Our study aimed to determine the potential association between miRNA processing gene polymorphisms and cervical precancerous lesions. Five single nucleotide polymorphisms (SNPs), including Ran-GTP (*RAN*) rs14035, exportin-5 (*XPO5*) rs11077, *DICER1* rs3742330, *DICER1* rs13078, and *TARBP2* rs784567, were genotyped in a case–control study to estimate risk factors of cervical precancerous lesions. The gene–environment interactions and haplotype association were estimated. We identified a 27% decreased risk of cervical precancerous lesions for individuals with minor G allele in *DICER1* rs3742330 (odds ratio (OR) = 0.73, 95% confidence interval (95% CI) = 0.58–0.92, *P* = 0.009). The AG and AG/GG genotypes in *DICER1* rs3742330 were also found to decrease the risk of cervical precancerous lesions (AG compared with AA: OR = 0.51, 95% CI = 0.35–0.73, *P* <0.001; AG/GG compared with AA: OR = 0.54, 95% CI = 0.39–0.77, *P* = 0.001). The GT haplotype in *DICER1* had a risk effect on cervical precancerous lesions compared with the AT haplotype (OR = 1.36, 95% CI = 1.08–1.73, *P* = 0.010). A two-factor (*DICER1* rs3742330 and human papillomavirus (HPV) infection) and two three-factor (model 1: rs3742330, passive smoking, and HPV infection; model 2: rs3742330, abortion history, and HPV infection) interaction models for cervical precancerous lesions were identified. In conclusion, the genetic variants in the miRNA processing genes and interactions with certain environmental factors might contribute to the risk of cervical precancerous lesions in southern Chinese women.

## Introduction

Cervical cancer accounts for approximately 15% of cancer-related deaths in women worldwide [1,2], and it is the second most common malignancy and third leading cause of cancer deaths amongst women in less developed countries [3]. There were an estimated 530000 new cervical cancer cases and 275000 deaths worldwide in 2015, and nearly 90% of these occurred in low- and middle-income countries [4].

Persistent infection with high-risk human papillomavirus (HPV) is widely recognized as the major risk factor of cervical cancer, based on abundant epidemiological and clinical evidence [5,6], and HPV infection is detected in approximately 99.7% of the cases [7,8]. There are more than 100 types of HPV, of which at least 12 high-risk types are carcinogenic to humans [9,10]. Nevertheless, high-risk HPV infection alone is not a sufficient factor to induce tumor progression. Less than 4% of HPV-infected individuals have persistent infection, and even fewer develop cervical cancer during their life [11,12], suggesting that other contributing factors, such as genetic factors, may also play a role in the pathogenesis of cervical cancer. There are a series of subsequent steps to develop cervical cancer: persistent HPV infection, development of precancerous lesions, and final progression to invasive cervical cancer, which will take up to 20–30 years [13–15]. Such a long period offers us some opportunities to reduce the incidence and mortality of cervical cancer by intervention, and early treatment prevents up to 80% of cervical cancers in Western countries [16,17]. The precancerous lesions can be treated timely, and cancer can be avoided by detection during screening for cervical cancer, which can effectively reduce the high mortality rate and huge medical expenses of cervical cancer [16].

MiRNAs are a class of short, noncoding, endogenous RNA molecules 18–25 nts in length [18]. Accumulating evidence has demonstrated that up to one-third of human genes are regulated by miRNAs [19], though miRNAs constitute at most 3% of the human genome [20]. Moreover, miRNAs can also act as tumor suppressors or oncogenes in human cancers, such as *miR-187* and *miR-27b* in cervical cancer [21,22], indicating that miRNAs are key regulators in both physiological and pathological mechanisms [18,23,24]. Several miRNA processing genes are required to complete the biosynthesis of mammalian miRNAs [24,25]. Initially, miRNAs are transcribed by RNA II polymerase into long precursors called pri-miRNAs, which are cleaved in the nucleus to release approximately 60–70-nt stem loop intermediate, known as the miRNA precursor hairpins (pre-miRNA). Then, Ran-GTP (*RAN*) and exportin-5 (*XPO5*) recognize and bind the pre-miRNA molecule, exporting it to the cytoplasm along a GTP to GDP gradient, and pre-miRNA is further diced into a short, double-stranded miRNA duplex by *DICER1*, co-operating with *TARBP2*. Finally, the duplex is unwound to form the mature miRNA, which interacts with mRNA to regulate gene expression [26–28]. *RAN* encodes a small G protein that is essential for the translocation of RNA and proteins through the nuclear pore complex, and the pre-miRNA export will greatly reduce if *RAN* is depleted [29,30]. *XPO5*, a member of the karyopherin β family, is related to the human export receptor that uses the GTPase Ran to control cargo association [31,32]. *TARBP2* is a dsRNA that encodes an integral component of a *DICER1*-containing complex [33], and *DICER1* is the key component of a highly conserved cellular pathway responsible for the generation of small RNAs, such as miRNAs and siRNAs [34].

It has been reported that the genetic variations in miRNA processing genes, including *RAN/DICER1/XPO5/TARBP2*, were related to many premalignant lesions and cancer diseases [35–39]; however, such evidence is lacking for the relationship between these miRNA processing genes and the risk of cervical precancerous lesions. Therefore, five polymorphisms in four candidate genes (*RAN* rs14035, *XPO5* rs11077, *DICER1* rs3742330/rs13078, and *TARBP2* rs784567) were genotyped. The association between the miRNA processing genes and cervical precancerous lesions in the southern Chinese population was investigated, and, we further assessed the potential effects of gene–environment interaction to the cervical precancerous lesions.

## Materials and methods

### Study participants

A total of 592 southern Chinese women were recruited in our study, ranging from 19 to 65 years (median age: 42.5 years). The study protocol was approved by the Ethics Committee of the School of Medicine at Jinan University. The face-to-face questionnaire survey was carried out to obtain the participants’ epidemiological data. And 5-ml peripheral venous blood sample from each participant was collected with the EDTA vacuum blood collection tubes and saved at 4°C, then transported to our laboratory in Jinan University. The anticoagulant peripheral blood was centrifuged at 2000 rpm for 10 min and the plasma layer was carefully drawn, then stored in the −80°C refrigerator. Cervical exfoliated cells from vaginal swabs were collected by cyto-brush (Qiagen, Valencia, CA) and preserved in 2.5 ml denaturation buffer. ThinPrep 2000 (Hologic Inc.) and SurePath liquid-based Pap test (BD, U.S.A.) were used for the ThinPrep cytological test. The cytological smear was read by three clinical cytopathologists, and the results were assessed according to the Bethesda System (2001) [40]. The normal cells were categorized as the controls and the abnormalities were defined as the cases, which consisted of high-grade squamous intraepithelial lesions (HSIL), low-grade squamous intraepithelial neoplasia (LSIL), and atypical squamous cells of undetermined significance (ASCUS). There were 296 healthy controls and 296 cases, including 44 HSIL, 120 LSIL, and 132 ASCUS.

### HPV testing

Both high-risk HPV (types 16, 18, 31, 33, 35, 39, 45, 51, 52, 56, 58, 59, 66, and 68) and low-risk HPV (types 6 and 11) were detected using the MassARRAY (Sequenom, San Diego, CA) technique based on MALDI-TOF MS. The total DNA of the cervical cells was extracted from the commercial magnetic beads kit (Chemagen, Pekinelmer, Waltham, MA) according to the manufacturer’s instructions. All procedures were performed in the clinical standard laboratory of BGI (Beijing Genomics Institute, Shenzhen, China).

### Selection of single nucleotide polymorphism and genotyping

The tagSNPs (single nucleotide polymorphisms) of miRNA processing genes were selected from the HapMap database (http://hapmap.ncbi.nlm.nih.gov) based on the criteria that the screened region covered most of the genetic information in the Han Chinese in Beijing (CHB) population, while the Hardy–Weinberg equilibrium (HWE) *P*-value, minor allele frequency (MAF), and *r^2^* threshold were 0.01, 0.01, and 0.8, respectively. The MAF of *TARBP2* rs784567 was less than 0.01, so this SNP was excluded when we analyzed the polymorphism associations between the cervical precancerous lesions and controls. Eventually, we selected four candidate SNPs in four miRNA processing genes as follows: rs14035 in *RAN*, rs11077 in *XPO5*, and rs3742330 and rs13078 in *DICER1*.

Peripheral blood samples were collected, and genomic DNA was extracted from the blood by the phenol-chloroform DNA extraction method. We genotyped the candidate SNPs by the MALDI-TOF MS method. PCR conditions and primers were designed by the MassARRAY Assay Design 3.1 software (Supplementary Table S1). Genotyping was performed in real time with Typer software version 4.0. Genotype sequencing was performed by BGI.

### Statistical analyses

The SPSS 21.0 software package (SPSS Inc., Chicago) was used for all statistical analyses. The characteristics between the cases and controls were compared using Student’s *t* test (for normally distributed continuous variables), Mann–Whitney U test (for non-normally distributed variable), and Chi-square test (for categorical variables). The rank correlation analysis was used to understand the expression correlation between mRNA and miRNA. HWE in controls was tested for each SNP, and the haplotype analysis was calculated using SNPstats (http://bioinfo.iconcologia.net/snpstats/start.htm). A logistic regression was used to calculate the odds ratios (ORs) and their 95% confidence intervals (95% CIs) for risk estimation. Possible biological interactions between HPV infection and SNPs were evaluated on an additive scale with 95% CI by calculating three measures: synergy index (S), attributable proportion due to interaction (AP), and relative excess risk due to interaction (RERI). If there is no biological interaction, the 95% CI of S is over 1, and RERI and AP cross 0.

Potential gene–environment interaction was analyzed using multifactor dimensionality reduction software 1.0.0 (MDR 1.0.0). The best model was determined by the testing balanced accuracy (TBA) and cross-validation consistency (CVC) indices. Statistical significance was defined as *P*-values <0.05.

### Quantitative real-time reverse transcription PCR analysis

We used the Blood RNA Kit (Omega Bio-Tek, Doraville, GA, U.S.A.) to extract the total RNA from peripheral blood samples, followed by reverse transcription using the transcriptase cDNA kit (Takara PrimeScript RT Master Mix kit, Otsu, Japan). We used a Bio-Rad CFX96 real-time system (Bio-Rad Laboratories) to perform the quantitative real-time reverse transcription PCR (qRT-PCR) analysis. The PCR conditions were as follows: 95°C for 30 s, followed by 40 cycles of 95°C for 5 s and 60°C for 30 s, then 95°C for 10 s, 60°C for 30 s, and finally 95°C. The mRNA expression was quantitated with the SYBR Primer Script RT-PCR kit (Takara, Otsu, Japan) and normalized by the expression of β-actin. 5′-ACTGCTGGATGTGGACCACACA-3′ and 5′-GGCTTTCCTCTTCTCAGCACTG-3′ were the primer pairs used for *DICER1*.

The miRNA was extracted from the plasma using miRNeasy Serum/Plasma Advanced Kit (Qiagen, catalog number 217204). All-in-One™ miRNA qRT-PCR detection system (GeneCopoeia, catalog number QP015) was used for the single-step cDNA synthesis and qPCR detection. The *miR-39-3p* of *Caenorhabditis elegans* (cel-*miR-39-3p*) was added to the sample as the reference for *miR-375* measurement. The PCR conditions were 95°C for 10 min, followed by 40 cycles of 95°C for 10 s and 58°C for 20 s, then the next step was at 72°C for 10 s. The qRT-PCR results were confirmed using the cycle threshold (*C_t_*) value, and the relative gene expression was calculated using the 2^−Δ*C*^*^t^* method: Δ*C_t_* = *C_t_*_(target gene)_ – *C_t_*_(reference gene)_. The results were expressed as the mean ± S.D., and the data between case and control groups were analyzed using the two-tailed Mann–Whitney U test, for which the significance level was set at *P* <0.05.

## Results

### Population characteristics

Table 1 shows the distribution of the general characteristics between the cervical precancerous lesion cases and controls. There were significant differences in age at first intercourse, number of pregnancies, and HPV infection. No significant difference was observed in other factors between the two groups.

**Table 1:** Demographic characteristics in cases and controls

Variables	Control, *n* (%)	Case, *n* (%)	T/χ^2^ value	*P*-value^1^
Age, yrs (mean ± S.D.)	43.10 ± 7.28	41.97 ± 8.45	1.74	0.082
BMI, kg/m^2^ (mean ± S.D.)	22.46 ± 3.14	22.14 ± 2.89	1.30	0.195
Age at menarche, yrs (mean ± S.D.)	14.95 ± 2.66	15.14 ± 1.86	−1.00	0.316
Age at first intercourse, yrs (mean ± S.D.)	22.96 ± 2.90	22.39 ± 2.98	2.34	0.020^2^
Age at primiparity, yrs (mean ± S.D.)	24.20 ± 3.29	23.94 ± 3.44	0.94	0.349
Number of pregnancies (mean ± S.D.)	2.54 ± 1.17	2.76 ± 1.36	−1.97	0.049^2^
HPV infection				
Negative	204 (68.92)	79 (26.69)	105.78	<0.001^3^
Positive	92 (31.08)	217 (73.31)		
Cancer family history				
Negative	290 (98.64)	295 (99.66)	0.82	0.365
Positive	4 (1.36)	1 (0.34)		
Gynecological history				
Negative	173 (58.45)	165 (55.74)	0.44	0.506
Positive	123 (41.55)	131 (44.26)		
Abortions history				
Negative	190 (64.19)	181 (61.15)	0.59	0.444
Positive	106 (35.81)	115 (38.85)		
Genital cleaning after intercourse				
Negative	129 (44.48)	106 (37.19)	3.16	0.075
Positive	161 (55.52)	179 (62.81)		
Passive smoking				
Negative	122 (41.22)	124 (41.89)	0.03	0.868
Positive	174 (58.78)	172 (58.11)		
Physical exercise				
Negative	220 (74.32)	219 (73.99)	0.01	0.925
Positive	76 (25.68)	77 (26.01)		

Data are shown using means ± S.D. for continuous fact. Abbreviation: BMI, body mass index; yrs, years.

*P*-values ^1^ were based on Student’s *t*test, Mann–Whitney U test, or Chi-square test.

^2^, *P*-value less than 0.05. ^3^, *P*-value less than 0.001.

### The association between SNPs in miRNA processing genes and risk for cervical precancerous lesions

All the SNPs agreed with HWE (Supplementary Table S2). A significant association was found between *DICER1* rs3742330 and a decreased risk of cervical precancerous lesions in the allelic model (G compared with A: OR = 0.73, 95% CI = 0.58–0.92, *P* = 0.009), suggesting that individuals carrying the G allele had a 27% lower risk of developing cervical precancerous lesions than those carrying the A allele. Individuals who carried AG and AG + GG genotypes in *DICER1* rs3742330 were at 0.51- and 0.54-times lower risks of developing cervical precancerous lesions, respectively, than individuals with the AA genotype (AG compared with AA: OR = 0.51, 95% CI = 0.35–0.73, *P* <0.001; AG + GG compared with AA: OR = 0.54, 95% CI = 0.39–0.77, *P* = 0.001); however, *RAN* rs14035, *XPO5* rs11077, and *DICER1* rs13078 were not associated with cervical precancerous lesions (Table 2).

**Table 2:** Association between SNPs in miRNA processing genes and cervical precancerous lesions

SNP	Model	Polymorphism	Control (*n*=296)	Case (*n*=296)	OR^3^ (95% CI)	*P*-value^3^
*RAN*rs14035						
	Codominant	CC	186	192	1.00 (Ref)	
		CT	95	93	0.88 (0.61–1.25)	0.464
		TT	15	10	0.69 (0.30–1.59)	0.386
	Dominant	CT + TT	110	103	0.85 (0.61–1.20)	0.356
	Recessive	CC + CT	281	285	1.00 (Ref)	
		TT	15	10	0.72 (0.32–1.65)	0.440
	Allele	C	467	477	1.00 (Ref)	
		T	125	113	0.89 (0.67–1.18)	0.400
*XPO5*rs11077						
	Codominant	TT	268	257	1.00 (Ref)	
		TG	27	37	1.58 (0.92–2.72)	0.096
		GG	1	2	1.75 (0.16–19.62)	0.650
	Dominant	TG + GG	28	39	1.59 (0.94–2.70)	0.086
	Recessive	TT + TG	295	294	1.00 (Ref)	
		GG	1	2	1.67 (0.15–18.67)	0.678
	Allele	T	563	551	1.00 (Ref)	
		G	29	41	1.44 (0.89–2.36)	0.139
*DICER1* rs3742330						
	Codominant	AA	90	134	1.00 (Ref)	
		AG	161	117	0.51 (0.35–0.73)	<0.001^2^
		GG	45	45	0.68 (0.41–1.12)	0.134
	Dominant	AG + GG	206	162	0.54(0.39–0.77)	0.001^1^
	Recessive	AA + AG	251	251	1.00 (Ref)	
		GG	45	45	1.00 (0.63–1.57)	0.989
	Allele	A	341	385	1.00 (Ref)	
		G	251	207	0.73 (0.58–0.92)	0.009^1^
*DICER1*rs13078						
	Codominant	TT	282	280	1.00 (Ref)	
		TA	14	15	1.05 (0.49–2.22)	0.902
	Dominant	TA + AA	14	15	1.05 (0.49–2.22)	0.902
	Allele	T	578	575	1.00 (Ref)	
		A	14	15	1.08 (0.52–2.25)	0.844

Abbreviation: NA, not available.

^1^, *P*-value less than 0.05.

^2^, *P*-value less than 0.001.

3 Adjusted for age at first intercourse and number of pregnancies in logistic regression models.

### Gene–environment interaction for cervical precancerous lesions

The two-factor interaction of gene and HPV infection was evaluated by three measures with their 95% CI, and an antagonistic additive interaction between *DICER1* rs3742330 and HPV infection was observed with an S less than 1 (S = 0.44, 95% CI = 0.22–0.85) (Table 3).

**Table 3:** Biological interaction of two factors between miRNA processing genes and HPV infection on cervical precancerous lesions

Factor	Measures		
	S (95% CI)	AP (95% CI)	RERI (95% CI)
rs14035 and HPV infection	0.63 (0.34, 1.16)	-0.47 (-1.21, 0.27)	-2.39 (-5.66, 0.88)
rs11077 and HPV infection	1.20 (0.48, 3.03)	0.15(-0.54, 0.84)	1.22(-5.36, 7.80)
rs3742330 and HPV infection	0.44 (0.22, 0.85)*	-0.92 (-1.97, 0.12)	-3.22 (-6.84, 0.41)
rs13078 and HPV infection	0.62 (0.15, 2.61)	-0.48 (-2.19, 1.22)	-2.33 (-8.19, 3.53)

* Statistically significant.

**Figure 1:**
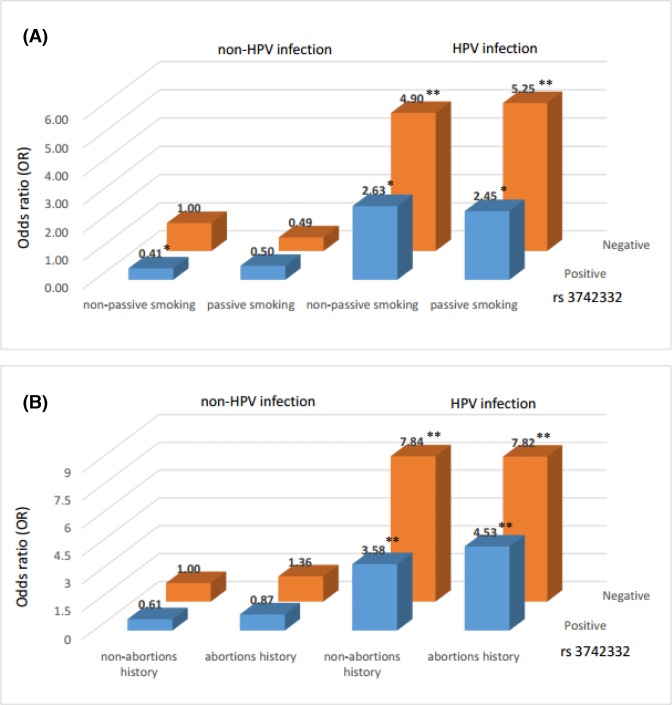
Risk analysis of gene–environment interaction (**A**) Model 1: *DICER1* rs3742330, passive smoking, HPV infection. The reference group is the combination of wild-type for rs3742330, non-passive smoking and non-HPV infection. (**B**) Model 2: *DICER1* rs3742330, abortion history, HPV infection. The combination of wild-type for rs3742330, non-abortions history and non-HPV infection is the reference group. The OR value is shown in the figure, *P<0.05 or **P<0.001 compared with the reference group.

To further explore the interaction effects of gene–environment on cervical precancerous lesions, we conducted MDR amongst the combination of *DICER1* rs3742330 and two other risk factors (Table 4). The two best interaction models were identified (model 1: *DICER1* rs3742330, passive smoking and HPV infection; model 2: *DICER1* rs3742330, abortion history and HPV infection) with the highest CVC value and a significant TBA value. We further conducted risk analysis of the three-way interaction among these two models (Supplementary Tables S3 and S4). As shown in Figure 1, the individuals with the combination of wild-type for rs3742330, passive smoking, and HPV infection in model 1 (OR = 5.25, 95% CI = 2.37–11.64) or the combination of wild-type for rs3742330, abortion history, and HPV infection in model 2 (OR = 7.82, 95% CI = 3.33–18.34) exhibited relatively higher risks of developing cervical precancerous lesions.

**Table 4:** MDR analysis for cervical precancerous lesions

Model	Interaction factors	TBA (%)	CVC	*P*-value
1	rs3742330, passive smoking, HPV infection	71.11	10/10	0.0011*
2	rs3742330, abortions history, HPV infection	71.11	10/10	0.0011*

* *P* value less than 0.05.

### Haplotype analysis

We performed haplotype analysis between the *DICER1* genes and cervical precancerous lesions (Supplementary Table S5). Compared with the highest frequency haplotype AT, the haplotype GT was significantly relevant to the risk of cervical precancerous lesions (OR = 1.36, 95% CI = 1.08–1.73, *P* = 0.010).

### Functional study

The expression of *DICER1* rs3742332 was tested from the randomly selected samples, with 38 controls and 35 cases; and we further selected 15 controls and 17 cases amongst them to measure *miR-375*.

**Figure 2:**
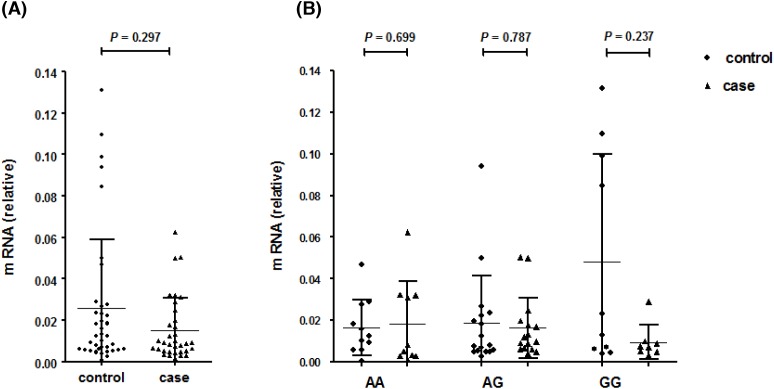
The mRNA expression result of *DICER1* rs3742332 (**A**) The relative mRNA expression in cases and controls of *DICER1* rs3742330. (**B**) The relative mRNA expression in cases and controls according to genotyping of *DICER1* rs3742330. The results were expressed as mean ± S.D. The qRT-PCR analysis of *DICER1* expression in 38 controls and 35 cases was compared using Mann–Whitney U test, and no statistical significance was found between cases and controls (*P*>0.05).

According to the qRT-PCR analysis, the relative mRNA expression levels of *DICER1* in the cases was lower compared with the controls, as shown in Figure 2A, but statistical significance was not reached (*P* = 0.297). In Figure 2B, there were no significant differences between the cases and controls in the subgroups of the three genotypes (both *P* >0.05).

The relative expression of *miR-375* in controls was slightly higher than the cases, but no statistical significance was found (*P* = 0.792) (Supplementary Figure S1). Then, we further assessed the correlation between *DICER1* and *miR-375* in the case group, the expression of *DICER1* mRNA and *miR-375* was positive correlation trend (Supplementary Figure S2), but no statistical significance was observed (*R* = 0.204, *P* = 0.433).

## Discussion

It has been established that miRNA processing genes are involved in the development of human cancers [35,37–39,41–43], but the roles of these genes in the pathogenesis of cervical precancerous lesions are still poorly understood. Therefore, we performed this case–control study to evaluate the relationship of SNPs in *RAN/DICER1/XPO5/TARBP2* and their interactive effects with environmental factors on the risk of cervical precancerous lesions in southern Chinese women.

We found that females carrying the variant allele G in *DICER1* rs3742330 were at a 0.73-fold decreased risk of developing cervical precancerous lesions compared with those with the A allele. *DICER1* is located on chromosome 14q32.13 and contains 1922 amino acids in humans, encoding an approximately 218 kDa RNase III endonuclease [23,44]. *DICER1* is a key component of a highly conserved cellular pathway, and it is responsible for the processing of gene-­encoded pre­-miRNAs into mature miRNAs [23]. Moreover, pre-miRNA and mature miRNA sequences will reduce if *DICER1* is silenced [45,46]. The polymorphic site rs3742330 A > G has been identified in the 3′-UTR of the *DICER1* gene, which might potentially influence the gene stability and expression, but the mechanism of rs3742330 remains unclear. It has been reported that the mutant genotype AG + GG of rs3742330 was associated with a decreased risk of gastric and colorectal cancer [35,41], and this gene variant has also been linked with protecting against the aggressiveness of prostate cancer [39]. Further, two previous studies reported that the variant genotype of rs3742330 is related to a higher survival of T-cell lymphoma patients [42,43]. Nevertheless, the rs3742330 polymorphic variant was correlated with a higher risk of the occurrence of laryngocarcinoma and oral premalignant lesions [37,38]. The inconsistent evidence from these findings suggests that *DICER1* may have various roles in the different diseases. According to our data, the individuals who carried the AG + GG genotype of rs3742330 had a 46% decreased risk of developing cervical precancerous lesions compared with those with the AA wild-type genotype (OR = 0.54, 95% CI = 0.39–0.77, *P* = 0.001, for the dominant model). Furthermore, an antagonistic additive interaction between rs3742330 and HPV infection was found. Taken together, the genetic variant G in *DICER1* rs3742330 was shown to have a potentially protective effect in cervical precancerous lesions.

In our qRT-PCR analysis, a relatively low mRNA expression of *DICER1* was observed in the cervical precancerous lesion patients compared with the controls, but there was no significant difference. Similarly, lower mRNA expression levels of *DICER1* were found in cervical cancer [47]; however, the mechanisms of its down-regulation in cervical cancer are still unclear. In addition, low expression levels of *DICER1* mRNA were also found to be associated with a poor prognosis and relapse of many diseases, such as cervical cancer [48], ovarian cancer [49], breast cancer [50], and lung cancer [51]. Thus, the reduced expression of *DICER1* appears to be a clinically useful prognostic factor for some cancers. These findings suggest possible mechanisms underlying low *DICER1* mRNA expression in not only cancer development but also prognosis and recurrence. *DICER1* is essential for the processing of pre-miRNAs to mature miRNAs, indicating that changes in *DICER1* might affect the synthesis and function of certain miRNAs. Previous studies reported that *DICER1* and *miR-375* were positively correlated, and *DICER1* might be involved in the feedback regulation of *miR-375*, which has been recognized as an important tumor suppressor [52]. Significantly decreased expression of *miR-375* was verified in both high-grade cervical intraepithelial neoplasia (CIN 2/3) and squamous cell carcinomas (SCCs) compared with normal cervical samples [53]. In our study, the relative expression of *miR-375* in controls was slightly higher than the cases, and the expression of *DICER1* mRNA and *miR-375* was positive correlation trend, however, no statistical significance was observed. Therefore, we could not determine the potential role of *miR-375*, as well as the relationship between *DICER1* and *miR-375* in this research.

To further explore the high-order, multiple-factor interaction of cervical precancerous lesion risk, we performed a risk analysis by MDR and found two best models. The *DCIER1* rs3742330 genotype and HPV infection, with the combination of passive smoking or abortion history, appeared to be correlated with the risk of cervical precancerous lesions. Passive smoking has been proven to be a risk factor of cervical precancerous lesions and cervical cancer [54–56]. Some researchers have found low levels of nicotine and cotinine in the cervical mucus of passive smokers [57–61]. Moreover, the combined effect of passive smoking and HPV infection might have an increased risk of SIL progression [56,62]. Therefore, we speculated that the variant of rs3742330 might have an interaction with HPV infection and passive smoking in cervical precancerous lesions. Until now, the association of LSIL and HSIL with a history of abortion had only been reported in Hindu women [63]. No previous research has confirmed the relationship between abortion history and cervical precancerous lesions, but any source of direct trauma to the cervix may be a potential risk.

In our study, no association was found between cervical precancerous lesions and *RAN* rs14035/*XPO5* rs11077/*DICER1* rs13078 in southern Chinese women. Future studies with larger samples are needed to determine these relationships. Similarly, Chen et al. [64] findings suggested that *DICER1* rs1057035 and *RAN* rs3803012 were not associated with cervical cancer in the Chinese population, and there are no previous studies that have investigated the association between polymorphisms in *RAN/XPO5/DICER1* genes and cervical precancerous lesions or cancer; however, rs13078 in *DICER1* was related to larynx cancer in a Polish population [38]. *RAN* rs14035 and *XPO5* rs11077 increased the risk of suffering from esophageal cancer in a Caucasian population [65]; however, *RAN* rs14035 CT heterozygotes were significantly associated with a reduced risk of colorectal cancer in a Korean population [66]. These studies suggested that polymorphisms in miRNA processing genes may play important roles in cancers.

The sample in our study was not very large, which may have weakened the statistical power. Additionally, this case–control study may include a selection bias, which may also affect the genetic associations, despite the adjustment for the confounding factors in the analysis. Therefore, a well-designed, larger study, and a more functional experiment should be conducted to validate our findings.

To the best of our knowledge, this is the first study to evaluate the relationship between miRNA processing genes (*RAN* rs14035/*XPO5* rs11077/*DICER1* rs3742330 and rs13078) and the risk of cervical precancerous lesions. Our results provide evidence that *DICER1* rs3742330 is associated with reducing the risk of cervical precancerous lesions in southern Chinese women, and our findings also suggest several possible interactions between *DICER1* genetic variations and environmental factors. Therefore, the present study provides new epidemiology clues about the roles of miRNA processing genes in cervical precancerous lesions.

## Supporting information

**Fig. S1:** The relative expression of miR-375 in 15 controls and 17 cases. The results were expressed as mean ± S.D. The qRT-PCR analysis of miRmiR-375 expression in controls and cases was compared using Mann-Whitney U test, and no statistical significance was found between cases and control (*P* = 0.792).

**Fig. S2:** The rank correlation analysis between DICER1 and miR-375 expression in 17 cases. There was a positive positive correlation trend (R = 0.204), but no statistical significance was observed (*P* = 0.433).

**Table S1:** Polymerase chain reaction primers and amplicon sizes

**Table S2:** General information for miRNA processing genes SNPs

**Table S3:** model 1: The interaction of risk estimates between rs3742330, passive smoking and HPV infection

**Table S4:** model 2: The interaction of risk estimates between rs3742330, abortions history and HPV infection

**Table S5:** The relationship between haplotype frequencies of rs3742330 and rs13078 in DICER1 and cervical precancerous lesions

## Abbreviations

AP: attributable proportion due to interaction
ASCUS: atypical squamous cells of undetermined significance
BGI: Beijing Genomics Institute
BMI: body mass index
*C_t_*: cycle threshold
CVC: cross-validation consistency
HPV: human papillomavirus
HSIL: high-grade squamous intraepithelial lesion
HWE: Hardy–Weinberg equilibrium
LSIL: low-grade squamous intraepithelial neoplasia
MAF: minor allele frequency
MDR: multifactor dimensionality reduction
nt: nucleotide
OR: odds ratio
PCR: polymerase chain reaction
pre-miRNA: miRNA precursor hairpin
qRT-PCR: quantitative real-time reverse transcription PCR
RAN: ran-GTP
RERI: relative excess risk due to interaction
S: synergy index
SCC: squamous cell carcinoma
S.D.: standard deviation
SIL: squamous intraepithelial neoplasia
SNP: single nucleotide polymorphism
TBA: testing balanced accuracy
XPO5: exportin-5
95% CI: 95% confidence interval
