# Supplementary material for: Association of variants of miRNA processing genes with cervical precancerous lesion risk in a southern Chinese population
Source: Biosci Rep. 2018 May 28;38(3):BSR20171565. doi: 10.1042/BSR20171565 (PMC6435547; doi:10.1042/BSR20171565)
Supplement: Supplementary file 1 [file bsr20171565_Supp1.pdf]

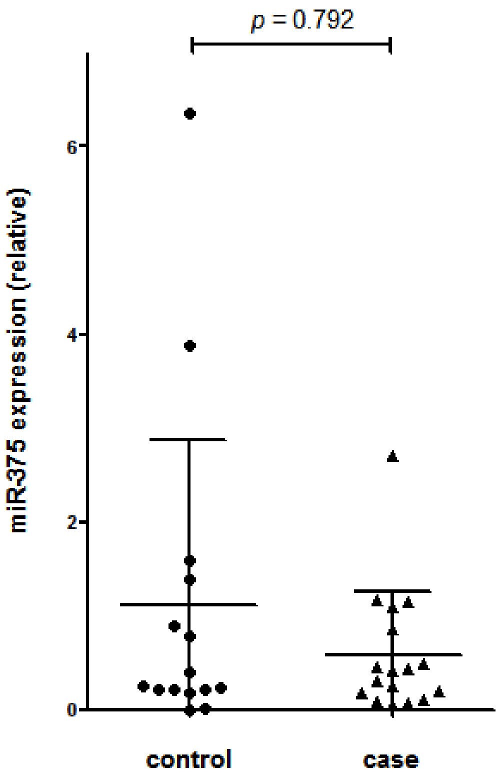

p = 0.792
miR-375 expression (relative)
6
4
2
0
control
case

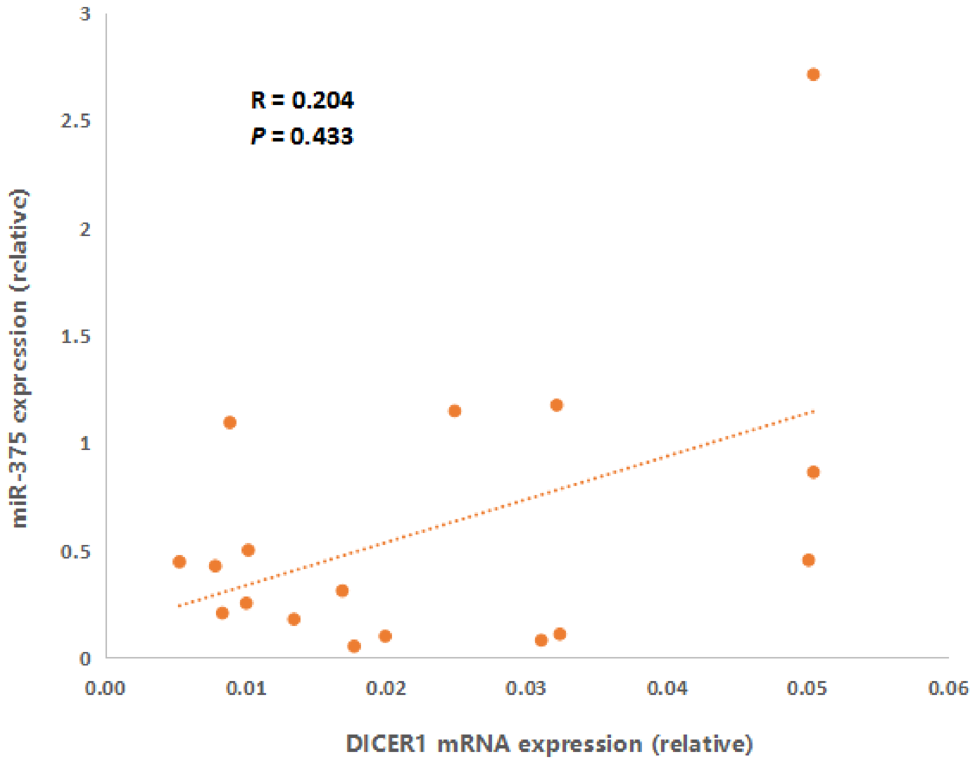
R = 0.204
P = 0.433
miR-375 expression (relative)
DICER1 mRNA expression (relative)
0
0.5
1
1.5
2
2.5
3
0.00
0.01
0.02
0.03
0.04
0.05
0.06

Table captions

Table 1 Demographic characteristics in cases and controls

Table 2 Association between SNPs in miRNA processing genes and cervical precancerous lesions

Table 3 Biological interaction of two factors between miRNA processing genes and HPV infection on cervical precancerous lesions

Table 4 MDR analysis for cervical precancerous lesions

Table S1 Polymerase chain reaction primers and amplicon sizes

Table S2 General information for miRNA processing genes SNPs

Table S3 model 1: The interaction of risk estimates between rs3742330, passive smoking and HPV infection

Table S4 model 2: The interaction of risk estimates between rs3742330, abortions history and HPV infection

Table S5 The relationship between haplotype frequencies of rs3742330 and rs13078 in DICER1 and cervical precancerous lesions

Figure captions

Fig. 1: Risk analysis of gene-environment interaction

(A) Model 1: *DICER1* rs3742330, HPV infection, passive smoking. The reference group in Fig.1A is the combination of wild-type for rs3742330, non-passive smoking and non-HPV infection. **(B)** Model 2: *DICER1* rs3742330, HPV infection, abortion history. The combination of wild-type for rs3742330, non-abortions history and non-HPV infection is the reference group in Fig.1B. The OR value is shown in the figure, *$P < 0.05$ or **$P < 0.001$ compared with the reference group.

Fig. 2: The mRNA expression result of *DICER1* rs3742332

(A) The relative mRNA expression in cases and controls of *DICER1* rs3742330; **(B)** The relative mRNA expression in cases and controls according to genotyping of *DICER1* rs3742330. The results were expressed as mean ± S.D. The qRT-PCR analysis of *DICER1* expression in 38 controls and 35 cases was compared using Mann-Whitney U test, and no statistical significance was found between cases and control ($P > 0.05$).

Fig. S1: The relative expression of miR-375 in 15 controls and 17 cases. The results were expressed as mean ± S.D. The qRT-PCR analysis of miR-375 expression in controls and cases was compared using Mann-Whitney U test, and no statistical significance was found between cases and control ($P = 0.792$).

Fig. S2: The rank correlation analysis between DICER1 and miR-375 expression in 17 cases. There was a positive correlation trend (R = 0.204), but no statistical significance was observed ($P = 0.433$).
